# Automated Specification-Based Testing of REST APIs

**DOI:** 10.3390/s21165375

**Published:** 2021-08-09

**Authors:** Ovidiu Baniaș, Diana Florea, Robert Gyalai, Daniel-Ioan Curiac

**Affiliations:** Automation and Applied Informatics Department, Politehnica University of Timisoara, Parvan 2, 300223 Timisoara, Romania; diana.florea@student.upt.ro (D.F.); robert.gyalai-korpos@student.upt.ro (R.G.)

**Keywords:** software testing, specification-based testing, automatic test case generation, REST API, OpenAPI 3.x

## Abstract

Nowadays, REpresentational State Transfer Application Programming Interfaces (REST APIs) are widely used in web applications, hence a plethora of test cases are developed to validate the APIs calls. We propose a solution that automates the generation of test cases for REST APIs based on their specifications. In our approach, apart from the automatic generation of test cases, we provide an option for the user to influence the test case generation process. By adding user interaction, we aim to augment the automatic generation of APIs test cases with human testing expertise and specific context. We use the latest version of OpenAPI 3.x and a wide range of coverage metrics to analyze the functionality and performance of the generated test cases, and non-functional metrics to analyze the performance of the APIs. The experiments proved the effectiveness and practicability of our method.

## 1. Introduction

Nowadays, in the era of the internet and interconnectivity, communication and interactions over web applications are very common. Web applications are not only interacting with their users through various devices, such as PCs, smartphones, and tablets, but they also exchange information with other applications. For these communications between applications to work properly, web services usually employ APIs as a backbone process. Hence, the high demand and usage of web services require high quality services that can be effectively ensured based on an automated and continuous testing process of the functionalities and performance of the APIs.

Soon after the release of the REST architecture [1], it became the most popular architecture used for Web APIs development. The reason behind this quick adoption is the fact that REST APIs are based on the HTTP protocol and make web applications easy to develop and maintain [2,3]. A study over the advantages of REST APIs [2] states that they are easy to use and comprehend, have a fast response time, and support a multitude of data types. It is worth mentioning that REST APIs are the most utilized API Architectural Style existing in ProgrammableWeb’s (https://www.programmableweb.com/news/which-api-types-and-architectural-styles-are-most-used/research/2017/11/26, accessed on 6 August 2021) APIs directory, with a utilization percentage of 81.53%. Furthermore, REST APIs will most probably continue being the most popular API architectural style in the following years [2]. 

The fact that REST APIs are preferred by both developers and businesses generates the need for continuous testing of APIs to maintain the quality and performance of the related web services. Automating the test case generation and execution upon REST APIs can be very efficient to the entire web service development and quality assurance. The resulting set of test cases, automatically generated based on the API specification, may cover a higher number of scenarios that would probably not be possible by manual test case creation. OpenAPI is the most common specification language for REST APIs [4] and version 3.x is the latest version released. Due to the major feature improvement compared to version 2.x, in the current work, we propose a test case generation process based on the latest OpenAPI version.

In the process of developing a specification-based solution for automated testing, the test case coverage metrics are arguably the most useful functional descriptors that can be analyzed to check how much of the system has been tested through the generated test cases [4,5,6]. In [7], a practical set of coverage criteria assembled in a comprehensive eight-level model, named “Test Coverage Model”, is proposed. The model offers different levels of coverage based on code coverage and faults detection measures. We employ these test coverage criteria to evaluate and demonstrate the effectiveness of our automated test case generation approach in real world settings. 

To summarize, this paper provides the following contributions:

- A novel technique to generate test cases for REST APIs based on black-box testing principles. This includes four types of configurations for generation of test cases, from fully automated test case generation to semi-automated test case generation with input from the user for certain parameters with no detailed values in the OpenAPI specification.
- Our approach mainly focuses on functional testing but includes the testing of one non-functional property of performance of the API. This non-functional metric is calculated as the average time for a response to be received after a request is sent.
- A wide range of coverage criteria analysis, by providing different types of coverage criteria based on the REST architecture and HTTP protocol properties in the OpenAPI specification. This approach aims to provide a more complex overview of the generated test cases for a better study of the validity of the test cases after they were executed.
- Our approach uses OpenAPI version 3.x, the latest OpenAPI specification.

The rest of the paper is organized as follows. Section 2 provides a theoretical background that we used to design and implement our approach. It also presents related work in the field of automated testing of REST APIs and underlines the specificities of our method. Section 3 describes the proposed method and its implementation. Section 4 offers a detailed and practical example of how the proposed procedure can be used to automatically generate test cases, together with some illustrative results that confirm the validity of the suggested methods. Section 5 provides discussion and limitations regarding our method. Finally, conclusions are drawn in Section 6.

## 2. Background

### 2.1. REST

**Figure 1:**
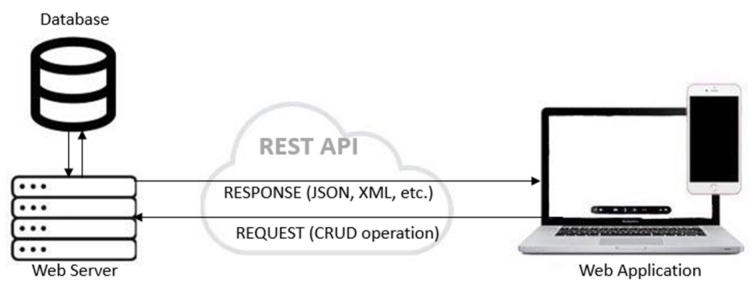
Applicability of REST API.

REpresentational State Transfer (REST) is an architectural paradigm employed to develop web services-based systems. Although REST is widely used when developing web services, it is not yet a standard. REST is using a combination of standards, such as HTTP, JSON, and XML, for describing its guidelines in developing HTTP web services. The main purpose of REST is to improve different non-functional properties of the web system, such as performance, scalability, simplicity, reliability, visibility, etc. Figure 1 presents the usage of REST API in the context of the communication between the client and a web service. The web application or another client makes a request to the web server for a resource. The request can be considered as formed by the REST API endpoint URL, API method—which mostly includes the CRUD operations (GET, PUT, POST, DELETE) and the parameters. The response is a representation of the resource, generally in the JSON or XML format. 

Status codes represent the type of responses received by the HTTP-based REST API calls. A status code is a threedigit number, the first digit represents the class (for example, 2xx—successful, 5xx—server error, etc.), the last two digits represent the specific response type (for example, 202—accepted, 401—unauthorized). They only represent a summary of the response and not the actual information that is requested. Status codes are very important and useful in the testing of REST APIs [8]. In our proposed solution, we focus on status codes to generate test cases that cover both successful and failed requests. In addition to this, we further use the information provided by the status codes to calculate the status code coverage metric and analyze the metric results. 

### 2.2. OpenAPI 3.0 Specification

OpenAPI (formerly known as Swagger Specification) is a widely used specification language for REST APIs [4]. With OpenAPI, the capabilities and functions of a web service are exposed without having access to the source code. Moreover, an entity can therefore understand how to interact remotely with the web service having minimal documentation about it written in the OpenAPI specification language [9]. Usually, OpenAPI defines the specification of the REST API, mostly in JSON or YAML formats. 

The OpenAPI architecture contains HTTP actions, such as GET, POST, PUT, and DELETE, and parameters which contain the name, type, format, and the location where the given parameter is used. Likewise, the OpenAPI specification contains a list of various responses represented by HTTP return codes [10].

The drawback of the OpenAPI specification language is that it does not cover the dependencies between the parameters. Parameter dependencies are common for web services as constraints could be applied on certain parameters to restrict their interactions or to require a combination of parameters to form a valid request [11]. There are a few versions of OpenAPI, the most used one being the OpenAPI 2.0 version, while the latest version available on the market is OpenAPI version 3.x [4]. After reviewing the API specifications available at APIs.guru (https://apis.guru/browse-apis/, accessed on 6 August 2021), we decided to employ the OpenAPI 3.x-based specifications to implement and validate our proposed solution.

### 2.3. Testing REST APIs

API testing is an important part of a software testing phase as this layer is closer to the user and it also connects the client to the server. API testing is very important to validate whether an API meets the expectations regarding reliability, performance, and security, as well as the specified functionality in various scenarios [4]. 

Testing a REST API can be challenging as it requires interaction with the requests and responses between the client and server entities [5]. It is as complex as the business service it exposes. The approach presented in [12] lists a series of principles that could provide a suitable strategy for testing an API. According to [13], web services testing can be split into six main stages: validation of the WSDL (Web Services Description Language), unit testing, functional testing, regression testing, load testing, and adherence to standards. In our proposed solution, we focus on the functional testing by validating that the communication between the server and the clients, and the requests and responses are behaving accordingly. 

In the current paper we address a real-life scenario: the information regarding the actual API implementation is limited to what is provided in the specification, thus restricting the number and types of test cases that can be implemented and executed. Taking into consideration the fact that the source code of the web application is not accessible, the API is black-box tested [5]. Consequently, functional testing is the most common type of black-box testing that can be employed. Black-box testing is also known as specification-based testing because the system under test is considered a black box with inputs and outputs. For specification-based API testing, the test cases are executed by sending HTTP requests to the API, and the responses of the API to the requests are analyzed and verified against the specifications [4].

Fault-based testing is a special type of testing technique that anticipates incorrect behavior of the system under test. It has the purpose of injecting incorrect values in the requests and to validate that the system will respond accordingly without failure. The fault-based testing can be applied when testing an API [4]. Requests with wrong values for the API parameters are sent on purpose, verifying whether the web service will return error status codes. In this situation, the test case will be considered successful if the system will not accept the wrong values.

Based on the currently mentioned principles and types of testing, our solution is designed to address both black-box testing and functional testing. We also employed fault-based testing for our test cases to cover more scenarios. 

### 2.4. Non-Functional and Functional Testing and Metrics over REST APIs

Each software is developed based on two types of requirements: functional requirements and non-functional requirements. Non-functional test cases are aiming to validate the system behavior against the written requirements and are addressing the following aspects: security, accessibility, performance, scalability, compatibility, reliability, usability [14].

Testing the non-functional properties of REST APIs is a challenging task since the APIs specifications provide little information about such properties. Based on our research, there are few software tools that can test non-functional properties of APIs, such as the tool presented in [15] and other online software tools, such as SOAP UI (https://www.soapui.org/, accessed on 6 August 2021) and Moesif API Analytics (https://www.moesif.com/solutions/track-api-program, accessed on 6 August 2021). These tools are mostly focused on measuring the performance and the availability of the operations described in the API specifications. We will also focus on a performance metric regarding the average duration needed for a request to be executed.

Compared to non-functional testing, the functional testing is easier to perform on REST APIs. We employ coverage metrics in our study, having the API requests as inputs and the API responses as outputs [7]. Consequently, the following coverage criterion types, theoretically presented in [7], are measured in the next sections:

- Input coverage criteria: path coverage, operation coverage, parameter coverage, parameter value coverage.
- Output coverage criteria: status code coverage, response body properties coverage, content-type coverage.

**Figure 2:**
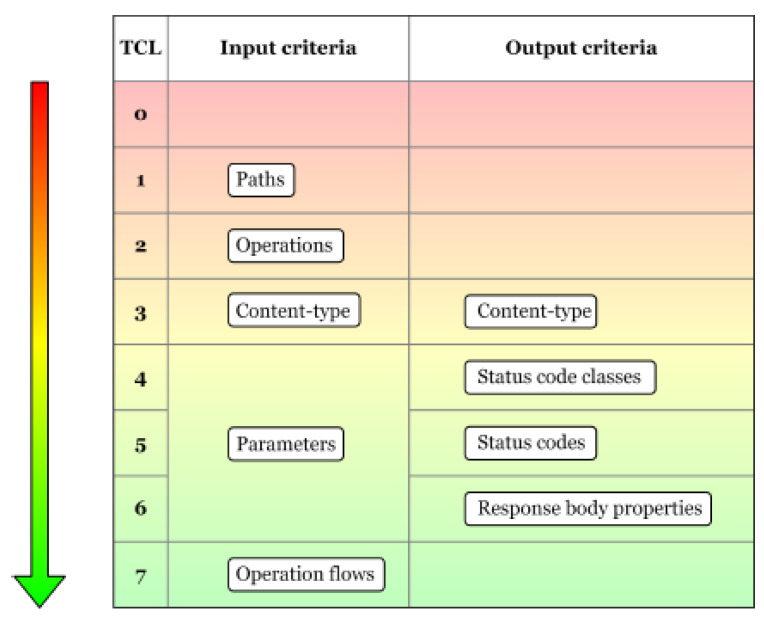
Test Coverage Model: Levels and Criteria [7].

According to [7], the coverage criteria, mentioned in the previous paragraph, can be arranged into eight test coverage levels (Figure 2), from the weakest coverage TCL0 to the strongest one, namely TCL7. To reach a specific level, all the previous coverage criteria from the other levels must be met. The role of this Test Coverage Model and its levels is to rank the test suits according to how much of the functionality is covered by the test cases. 

## 3. Related Work

Analyzing the scientific literature, we have found several approaches and methods of testing web services by using the project specifications to generate test cases. Many of these works were focused on web APIs developed using the SOAP standard. Approaches such as [16,17] are generating specification-based test cases focusing on the WSDL definitions of the SOAP Web APIs. The method presented in [17] focuses on fault-based testing, which is one of our testing approaches. In [16], the test case generation based on specifications is mainly focused on data types of different parameters and on operations that can be applied to API parameters. The approach presented in [16] is similar to our approach, as we also focus on testing parameters based on their data types and on testing various operations performed over the API parameters. 

In recent years, a larger number of methods have been proposed for automation of testing and generation of test cases for REST APIs. These are either black-box or white-box approaches. Arcuri proposes EvoMaster [18], an automated white-box testing approach which generates test cases using an evolutionary algorithm. In this approach, the API specification is based on OpenAPI, similar to our approach. However, being a white-box testing solution, it requires access to more than just the API specification, but also to the source code. In the case of [18], in order for the solution to work it requires access to the Java bytecode of the API. In further work, Zhang et al. [19] develops the previously mentioned EvoMaster into a more complex solution for resource-based test case generation by adding templates with organized test actions and using a search-based method to generate test cases.

Black-box testing techniques over APIs are employed when access to the source code is not possible and, therefore, test case generation can be automated based only on the API specification. Chakrabarti and Kumar [20] propose an automated generation of test cases for black-box testing (Test-The-Rest) where a manual definition of the model is required. Another method that is worth mentioning employs an open-source software tool called Tcases to generate test cases from the API specification [5]. Similar to our proposed solution, the test cases are generated from the OpenAPI specification and the method that generates the test cases involves combinatorial testing (i.e., both valid and invalid values are considered). There is a significant difference between our proposed solution and the one presented in [5]: while Tcases generates the test cases in a model-based manner to a minimal coverage of the requirements, our solution uses combinatorial and domain testing principles to generate test cases, so that the variety of test cases covers as many scenarios as possible. RESTler [21] approach is similar to our approach in the sense that both use status codes as a major factor in testing results. Whilst RESTler aims to be a security testing tool with the goal of finding faults in the REST API, we instead aim to test different aspects of the REST API, not only its faults. 

Ed-douibi [4] proposes another method of generating specification-based testing. Our approaches are related in many aspects, such as OpenAPI being the API specification source, as well as using fault-based testing. In addition, our proposed solution introduces four different configurations that allow the user to choose what kind of test cases to be generated. The Standard configuration is the simplest and most related to [4] and was used as the starting point for our solution. The other three configurations contain all of the available parameters from the specification that require an initial input from the user to generate test cases that depend on parameters that are not specified or required. Besides this, we further developed the single test coverage criteria used in [4] by analyzing different types of coverage for the tests that were executed. The OpenAPI version used by Ed-douibi [4] is OpenAPI version 2.0. We focused on the newer version of OpenAPI 3.x, that is growing in usage and comes with updated document structure, including new and reusable component objects and improved JSON schema. Karlsson [10] proposes QuickREST, a solution similar to ours but focusing on property-based testing. 

Related to the non-functional properties testing of REST API, there is a limited number of approaches developed in this field. There are some commercial tools, such as SOAP UI (https://www.soapui.org/, accessed on 6 August 2021), which propose testing non-functional properties of APIs. However, they mostly focus on load testing. In [15], a framework named GADOLINIUM is presented. It automatically tests non-functional properties, such as performance and availability of the API. Influenced by this approach, we decided to include the non-functional performance metric beside our other functional metrics, mainly to study if performance is impacted by the different configuration in which we generate tests for an API. 

The previously mentioned approaches are focusing on test case generation via API specification, as well as execution of test cases to measure the test coverage criterion. Nevertheless, we consider the test coverage of these approaches could be improved. Hence, our solution comes with a novel approach and a detailed report on multiple test coverage metrics. Moreover, our solution provides both fully automated and semi-automated generation of test cases through the four configurations. Therefore, the user has a wider range of choices for the types of test cases to be generated which makes our solution different from the literature that has been reviewed. In addition, our approach also tries to shift the focus to a non-functional metric, such as performance. Based on the literature review, to the best of our knowledge, our proposed solution is the first that combines testing and measuring of both functional and non-functional properties of a REST API.

## 4. Proposed Approach

The purpose of our approach is to validate any REST API by automatically generating and executing test cases based solely on its OpenAPI specification. Since we have no information regarding the way the API was implemented, we follow the black-box testing principles. To make the procedure of test generating more versatile, we proposed to include different levels of configuration that can be chosen by the user. For the purpose of having detailed results, we decided to include several coverage criteria as well as a performance metric when creating the test statistics for an API. The following subsections will present the proposed method, the four types of testing configurations that can be selected, and an in-depth description of the actual implementation.

### 4.1. Method Description

**Figure 3:**
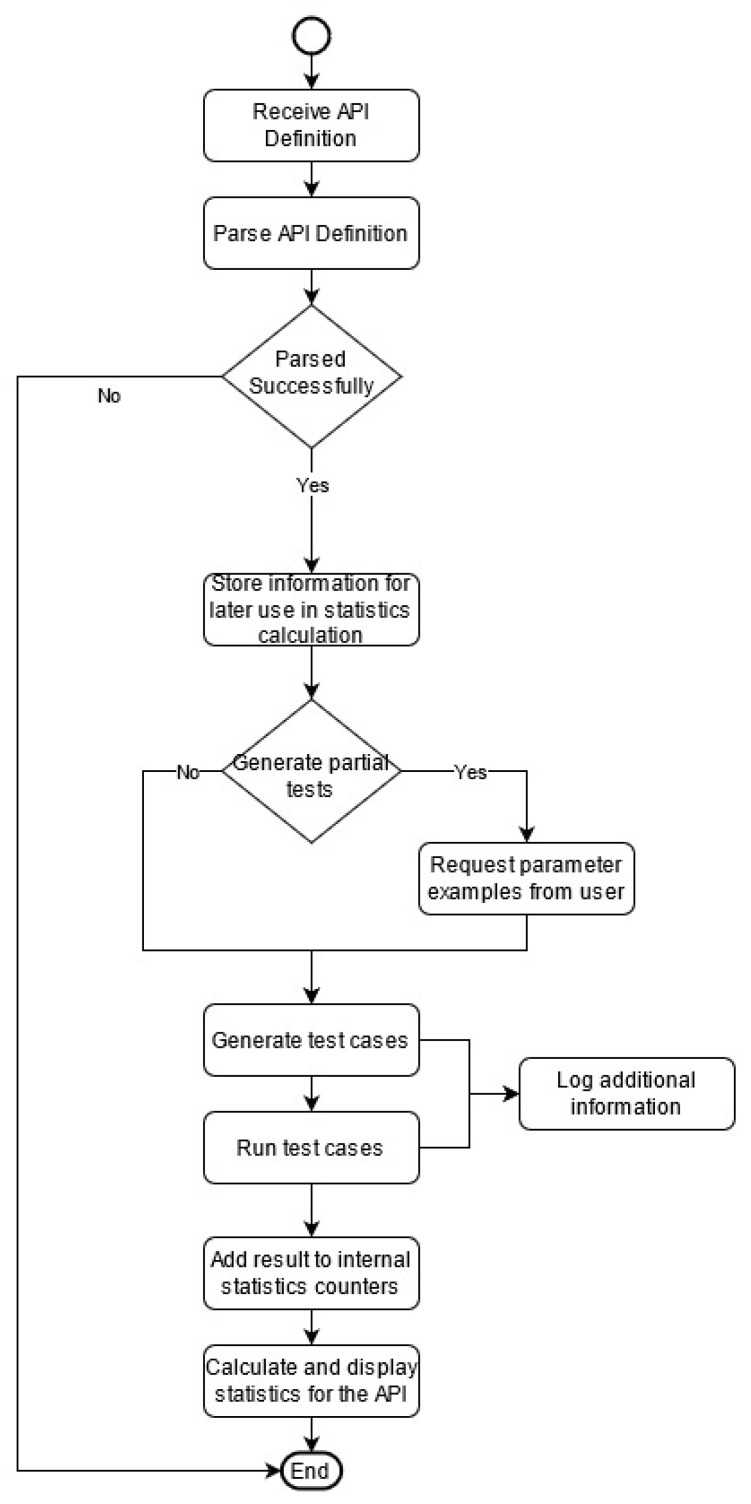
System activities and actions (activity diagram).

Our proposed solution requires either the definition of the API under test or the link to the definition of the API in JSON format as input. This definition is then interpreted by the system. It will output a collection of specifically designed objects that will be used in our implementation, as well as execution, to encapsulate the information contained in the API’s definition. Such an object includes information about the number and type of parameters, the path, and the type of an operation, etc. A flow diagram of our proposed solution, including the activities and actions of our system, is presented in Figure 3.

**Figure 4:**
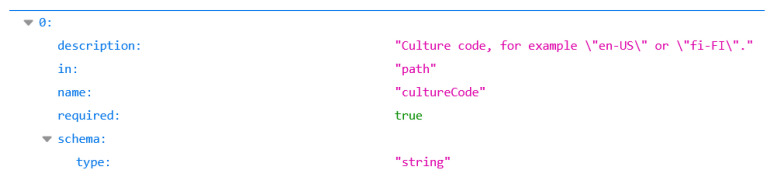
Descriptive values for API parameters.

Based on the pre-runtime settings, there are different types of test cases that our proposed test case generator system can provide:

1. Test cases with minimum number of parameters: Only the parameters that are specified as “*required*” in the OpenAPI specification of the API under test. *Required* is a Boolean property type which, in this case, must have the value *true*. If the property does not appear in the specification, then it is considered with the value *false* by default [9].
2. Test cases with maximum number of parameters: All the parameters that are defined in the OpenAPI specification will be used for generating the test cases. The *required* property will be ignored.
3. Automated test cases: No input from the user is required, the test cases are automatically generated.
4. Partial test cases: The user is required to give an input only if the information that is needed is not in the specification or API definition. When a request is made for the user to input some information, the software will require the name of the parameter, the data type, and, optionally, its description. Usually, sample data or other information is provided in the definition of the parameter regarding its values, although this information is not explicitly written in the specification. Such a case is presented in Figure 4, where the parameter *cultureCode* does not have the *example* attribute defined in the specification, therefore the standard implementation would send a random string that would not be accepted by the API. Hence, the user will see the information regarding the parameter that is provided in the specification and will know what kind of input is expected. Once the software receives the input from the user, the test case will be automatically generated.

**Figure 5:**
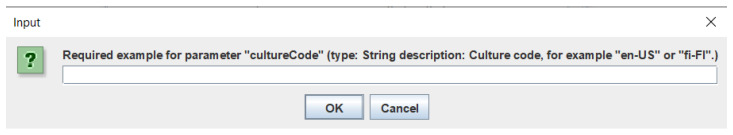
User input dialog example.

In the given example, the user would be presented with a dialog such as the one in Figure 5. The input from the user is then used in the test generation process as a valid value, replacing the usual defaults. Thus, all the operations that make use of this parameter are more likely to behave correctly and, in turn, make it easier to detect actual problems with the API. Given the fact that one parameter may be used in different operations, the input of the user is cached and reused if necessary.

To generate the test cases, each parameter will have a series of correct and erroneous values depending on the parameter type [4]. When the API specification provides parameters with values ranging in a given interval, we apply the principles of domain testing. A test case will include the execution of a particular request with all possible combinations of parameters. To determine if a test passed or failed, it will be verified if the values of all parameters were according to the specification provided for their specific category. In the case in which all the parameters have correct values, according to the specification, a successful response with status code of 2xx will be expected. On the other hand, when performing fault-based testing, an error response will be expected if at least one parameter is given an incorrect value. 

While the test cases are being executed, data is collected and stored for later use. At the end of the execution of all the test cases for the given API, the metrics will be calculated and the statistics regarding coverage and API performance will be displayed. 

A combinatorial function will be applied based on the number of parameters necessary to perform a request. In the case the number of possible combinations is higher than a given threshold, a specific function and method will be used to generate a suitable combination of parameters. 

Given the sequence of p parameters for an API operation $\left\{x_{1},x_{2},\ldots ,x_{p}\right\}$, a subset $V_{i}=\left\{v_{1},v_{2},\ldots \right\}$ of values for each parameter $x_{i}$ will be generated based on the parameter type. Consequently, we generate the following number of test cases:

$$\sum_{i=1}^{p}\left(\mathrm{card}(V_{i})-1\right)+1$$

Any parameter $x_{i}$ will take in turn all its possible values in the set $V_{i}$, while the rest of the parameters will have the same value. When the total number of combinations is less or equal to the given threshold, all the possible combinations will be used. The resulting number of combinations will be:

$$\prod_{i=1}^{p}\left(card(V_{i}\right)).\;$$

### 4.2. Implementation Details

In this paragraph we will describe our Java implementation using Eclipse IDE based on the classes, explaining classes functionalities and their roles. We used Maven to handle all our dependencies, one of them being the Jersey plug-in for making the needed requests to the API, as well as Google’s JSON-Simple plug-in to facilitate easier handling of the JSON inputs. When approaching this implementation, we aimed a high degree of decoupling between the components to facilitate easier expansion or maintenance. The main components are the user interface, the utility classes as configurations and logging, and the business logic that generates and runs the test cases based on the given specification. The rest of this section will elaborate more on the core/business logic implementation. 

**Figure 6:**
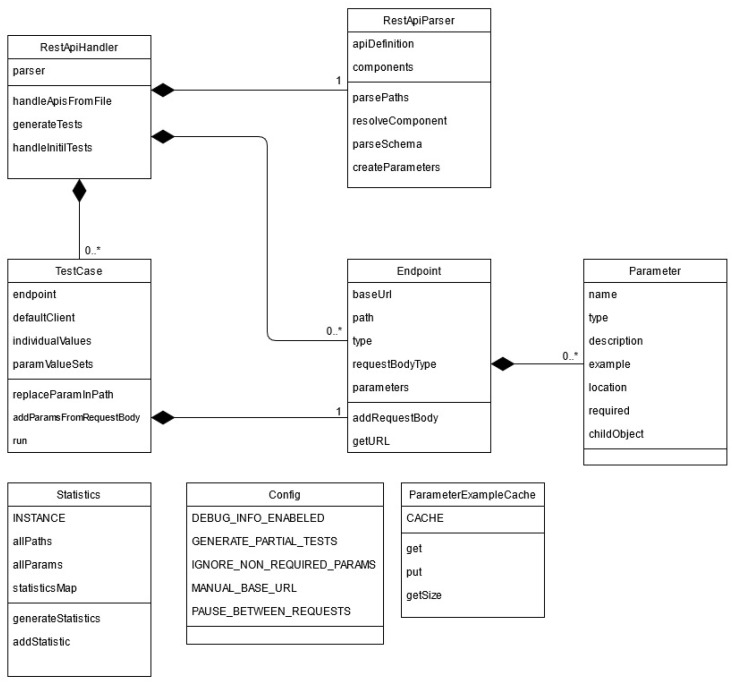
Structural description of the system (class diagram).

The main part of the program can, in turn, be divided into two sections: the parsing of the specification and the generation and execution of the test cases. The class diagram of our proposed solution is presented in Figure 6. The functionality and implementation of the classes presented in Figure 6 will be further detailed in the following paragraphs. 

The *ApiHandler* class receives the URL or file which contains the API definition and then executes the process by making use of the other classes. The *ApiParser* reads and parses the API definition and, when the process is over, outputs a list of *Endpoint* objects that encapsulate all the information that will be necessary in the next stages of the implementation. An *Endpoint* object represents an operation. For each *Endpoint* object, a *TestCase* object is created and all the test cases associated with the operation will be executed. The statistics are calculated and displayed.

*ApiParser* class receives the API specification as an input in JSON format. From given specification, it extracts all the data needed to build the *Endpoint* objects and the *Parameter* objects and stores the relevant statistical data. An example of data that is stored is the total number of parameters and the total number of operations. To aid the parsing of the information from the JSON file, an external library called JSON-Simple (https://mvnrepository.com/artifact/com.googlecode.json-simple/json-simple, accessed on 6 August 2021) was used. 

Both *Endpoint* class and *Parameter* class have the purpose of memorizing all the data that is needed to generate and run a test case for a particular operation. An *Endpoint* contains data such as the base URL, the path, the type of the operation, as well as a list of all parameters needed for that particular operation. Each *Parameter* in this list contains data such as name, type of parameter, location, etc.

*TestCase* class receives an *Endpoint* as an input parameter, for which it will later build a test case scenario. The first step of creating this test case scenario is assigning a set of values to the parameters present in the respective *Endpoint.* Both valid and purposefully erroneous values will be given to the parameters as we are integrating a fault-based testing approach. This process can be influenced by a series of factors:

- Depending on the chosen configuration, all or only the required parameters will be taken into account.
- The data type of each parameter will determine the default valid and invalid values that are given to the parameters.
- When the parameter is a numeric data type and it has a specific range defined in the specification, the given values respect the domain testing principles.
- If an example is given in the specification or if an example was provided by the user (given that the appropriate configuration is selected), that value will overwrite the default valid value for the parameter.

After each parameter has its set of values assigned, a combinatorial function is used to generate all the possible permutations (or a subset of them if the total number is over a set threshold). Each one of these combinations represents a request that will be made to that endpoint of the API. Once the test case scenario is generated, all the requests will be executed sequentially. In order for the execution to take place, an external library called Jersey-Client (https://mvnrepository.com/artifact/org.glassfish.jersey.core/jersey-client, accessed on 6 August 2021), was used. During and after this process, all the relevant information will be memorized for the statistics.

*ParameterExampleCache* is used only when the user is requested to give an input. The purpose of this class is to cache a parameter in case it is used in several requests. 

*Config* is a static class which provides the values that were previously configured. 

*Statistics* class has the purpose to calculate the statistics for the API under test based on all the data resulted from the execution of the test cases.

In terms of performance, our program utilizes only a small part of a computer’s resources, and it can function in parallel with other programs. RAM usage varies from 70 to 150 MB (results may vary depending on the complexity of the API and the chosen configuration) and the CPU usage peaked at 7% during a run (running on an Intel i7 CPU). As our program is self-contained, it only communicates with the API directly, so there is no possibility of interference with other programs. 

The implementation described above that was used to test and obtain the results that will be discussed in the following chapters can be found on GitHub (https://github.com/RobertGyalai/RESTApiTester/, accessed on 6 August 2021).

## 5. Results

In the following section, we offer two detailed case studies showing how our program performs. In the first part, we take into consideration a complex and illustrative API and we evaluate different types of coverage criteria such as path coverage, operation coverage, parameter coverage, parameter value coverage, content-type coverage, status code coverage, response body properties coverage, and content-type coverage. In the second part, our approach is tested and then analyzed over 30 different APIs, providing an overview of the statistical test case execution results. 

### 5.1. First Case Study: “College Football Data” API Testing

“College football data” API (https://api.apis.guru/v2/specs/collegefootballdata.com/2.2.14/openapi.json, accessed on 6 August 2021) gives access to a variety of data regarding college football. We have selected this API to evaluate our solution because it provides a wide range of data and it exposes a very good number of paths (42 paths) and parameters (38 parameters), as well as a well-defined and complex OpenAPI 3.0 specification.

The proposed solution was evaluated in four different configurations:

1. *Standard*: using a minimum number of parameters (only those required by the API) and with no generation of partial test cases.
2. *Max Parameter Coverage:* all parameters were used, either required or not required, with no generation of partial test cases.
3. *Partial Test Generation:* using a minimum number of parameters, only the required ones, with generation of partial test cases.
4. *Max Parameter Coverage and Partial Tests:* using all parameters (required or not required) with generation of partial test cases.

It is worth mentioning that an API specification defines multiple paths, each having one or more operations as *GET* and *POST*, while every operation represents a functionality of the API and each of them may require one or more parameters to be given when making the request. Running the Partial Test Generation configuration, there were nine parameters that needed to be set by the user, while for the Max Parameter Coverage and Partial Tests, 37 parameters needed to be configured. 

The four mentioned configurations generated the following number of test cases: Standard—75 test cases; Max Parameter Coverage—250 test cases; Partial Test Generation—78 test cases; and Max Parameter Coverage and Partial Tests—272 test cases.

In the following paragraphs, we will investigate the statistics provided by our implementation according to the Test Coverage Model: Levels and Criteria [7] that is briefly presented in Figure 2.

#### 5.1.1. Path Coverage

Path coverage metric refers to the number of API paths taken into consideration during a run, out of the total number of paths available from the API specification. This metric belongs to the level TCL1 [7] which represents, according to Figure 2, the lowest level of coverage. In our chosen API example, there were a total of 42 paths defined (such as */plays*, */teams*, */venues*, */games/teams,* etc.). Due to our approach, differentiating between parameters with or without parameter examples is important, thus we propose splitting this metric into two categories, path covered with full examples and paths covered without full examples. These two categories are based on whether or not all the parameters defined for a specific path have examples given in the API description. 

In our approach, we cover all the paths, even if they fall in the second category. Hence, we were able to run test cases for most of the paths in the specification, the exception being that we could not test operations of type *DELETE* and *PATCH*, because they require a prior knowledge of the available API data.

**Figure 7:**
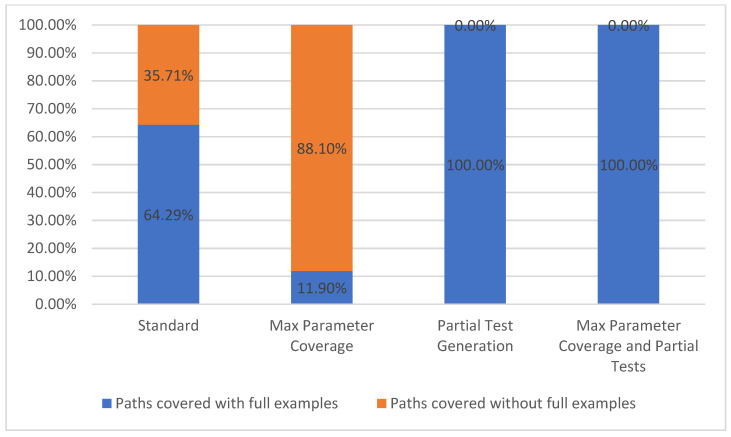
Path coverage.

In the *Standard* configuration, 27 out of the 42 paths were covered with full examples, while the remaining 15 were covered without full examples. When using the *Max Parameter Coverage* configuration, there were more parameters without examples that were being used in each path, thus making the total number of paths covered with full examples drop to five out of forty-two. For the last two configurations that include the partial test feature enabled, all the parameters that were previously without examples received the according values due to the user interaction. Thus, the paths covered with full examples now have 100% coverage. The equivalent statistics are presented in Figure 7. 

#### 5.1.2. Operation Coverage

Using the same reasoning as mentioned above, we have chosen to split the operation coverage metric into two parts based on whether or not all their parameters contain examples: operations covered with full examples and operations covered without full examples. Operation coverage metric belongs to Test Coverage Model TCL2 [7]. 

**Figure 8:**
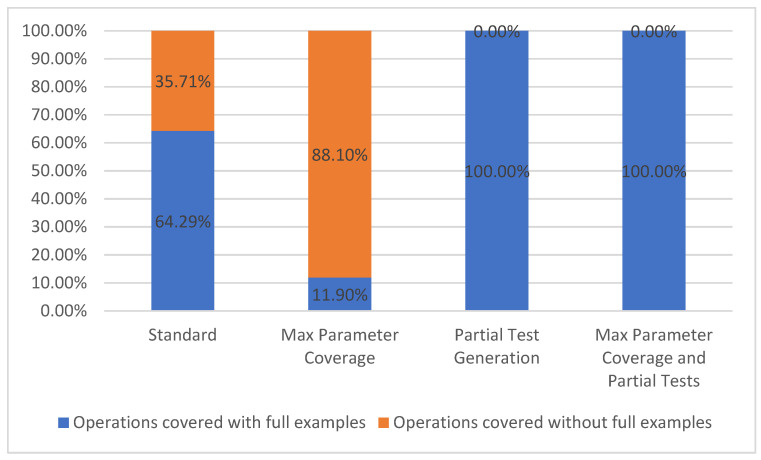
Operation coverage.

As previously mentioned, an API path can have one or more operations associated with it. In the current case study, every path had one GET operation, resulting in a total of 42 operations considered. The results presented in Figure 8 are the same as the results for path coverage due to the fact that there is only one operation for each path. 

#### 5.1.3. Paths Tested

There are two categories for tested paths: paths tested with success and paths tested with failures. When testing a path, there can be one or more requests that are made to the API depending on the number of parameter combinations. Each one of these requests will have a result based on the status code class. A request is considered successful if it has received a response with a 2xx status code (for example, the path */metrics/wp/pregame* returns a successful response code of *200* along with the actual response which, in the current case study, represents the win probabilities for a game). A request is considered to have failed if we receive a response with a status code different from the 2xx status code class (for example, *404*). For the handling of the test cases that use intentionally invalid parameters (applying the fault-base testing principle) the comparison is reversed. It is considered a successful test if the API returns an error status code, and the test is considered a failure if the API return a 2xx status code (meaning that the API accepted the invalid value).

**Figure 9:**
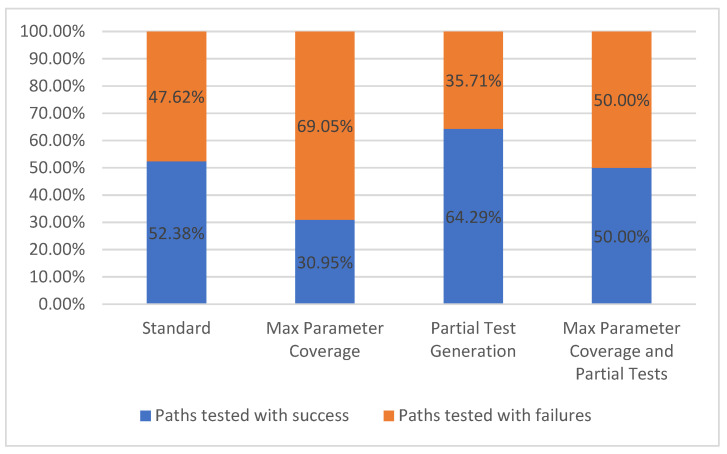
Paths tested.

Each set of parameter combinations will have an expected outcome for that request. A path is considered as tested successfully if all the responses match the expected ones. Otherwise, the tested path is considered a failure. The results for this metric are presented in Figure 9. 

It can be observed that once the number of parameters is rising, the number of passing test cases is decreasing due to the higher probability of errors and due to a higher number of parameters without examples. In the current situation, the GET operation for the */metrics/wp/pregame* path has four parameters but none of them are required. Thus, in the *Standard* configuration the request is undertaken with no parameter, while all the parameters were given values in the *Max Parameter Coverage* configuration. 

In the last two configurations, *Partial Test Generation* and *Max Parameter Coverage and Partial Tests*, which have partial tests enabled, we observe a growing number of successful test cases because of the user interaction which provides valid parameters for the requests based on the operation description. For example, the parameter *seasonType* from the GET operation is of type String, has no example, but has *“regular or postseason”* as its description. The written description is simple to interpret by the user that will consequently input a correct example, while the default string value that was given to the parameter in the two previous configurations, would not produce valid samples. 

#### 5.1.4. Operations Tested

**Figure 10:**
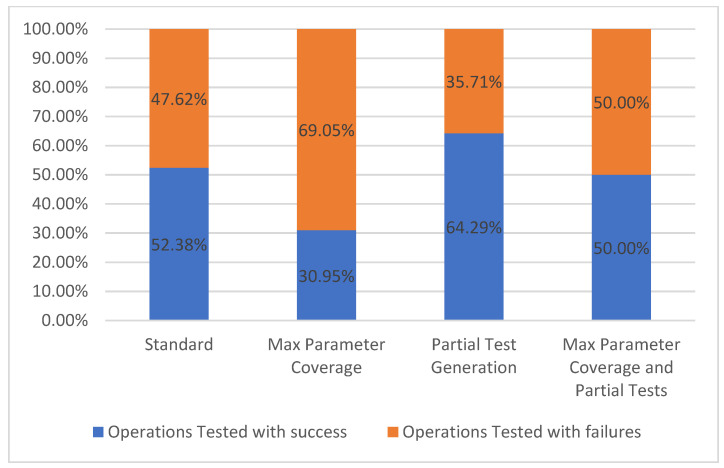
Operations tested.

The operations tested metric follows the same rules as the path tested metric, however the difference is that only individual operations are taken into consideration. As mentioned before, for the selected API, each path has only one operation, therefore the results presented in Figure 10 are the same as the previous metric. 

#### 5.1.5. Parameter Coverage

For a parameter to be considered as covered, it needs to have been used in one or more test cases. We separated the covered parameters into two groups based on the overall results of the test cases generated for each operation. The two groups are represented, on one hand, by the parameters covered in successful test cases and, on the other hand, by the parameters covered in failed test cases. These two groups form the parameter coverage metric, which corresponds to levels TCL4–TCL6 [7] presented in Figure 2. 

**Figure 11:**
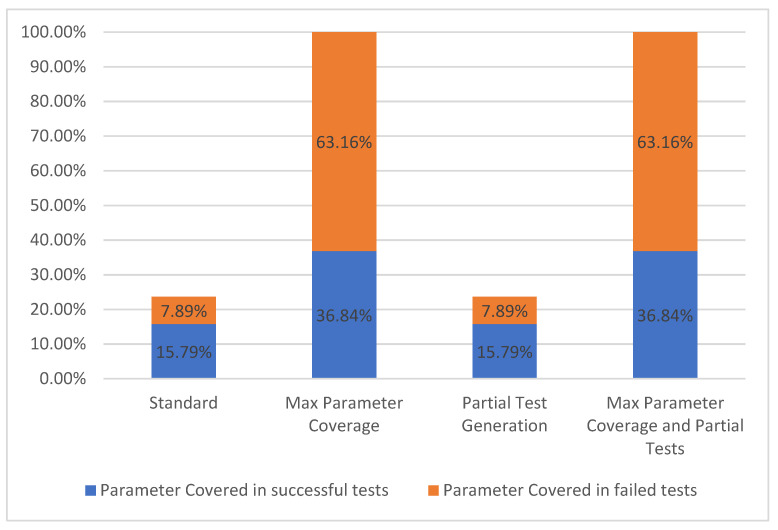
Parameter coverage.

The results concerning the parameter coverage metric are presented in Figure 11.

In the *Standard* configuration, only the required parameters were populated with values. The selected API had nine required parameters out of thirty-eight. Using the second configuration *Max Parameter Coverage*, all of the 38 parameters were utilized but only 14 of them were present in successful tests. 

For the “College football data” API, we observed that the configurations, which include partial tests, have no significant impact on this coverage metric, although the activation of the partial tests options actually increased the number of operations tested with success. In this case, the additional operations that were successful did not include parameters that were previously covered in the failed tests. Even if a parameter has a valid example provided by the user, it can still be part of an operation which has failed test cases because of other API related circumstances. Consequently, that specific parameter is still not counted as covered in successful tests. 

#### 5.1.6. Status Code Coverage

**Figure 12:**
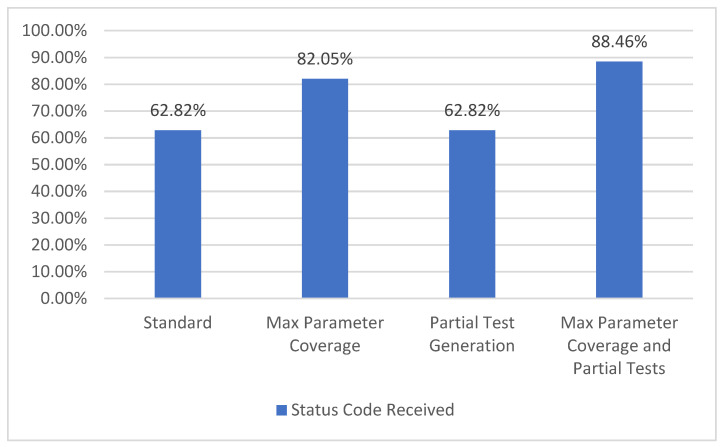
Status code coverage.

In the API specification, there may be one or more possible responses defined for a given operation. One of the attributes for this type of responses is the status code. The GET operation for the */metrics/wp/pregame* provides two possible response codes, *200* and *400*. Consequently, we compute the percentage of each returned status code. The resulting percentage is the status code coverage metric which corresponds to level TCL5 [7], as presented in Figure 12. 

In the selected API, there are a total of 78 possible status codes defined. With the *Standard* configuration, 48 status codes are covered, while with the *Max Parameter Coverage* configuration, 64 status codes are covered. In the third configuration it can be observed that there is no increase in the percentage, compared to the *Standard* one, as the few extra parameter examples provided by the API do not have an influence over this metric. However, in the last configuration with more parameters and more examples/input from the user, the resulting percentage for this metric is the highest of all four configurations. 

#### 5.1.7. Content Type Coverage

**Figure 13:**
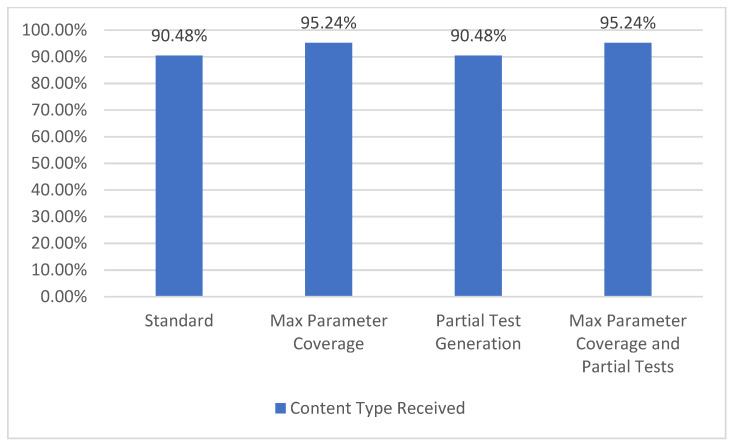
Content type coverage.

The API specification may include one or more responses for each operation and some of these responses can define the specific content type that is expected to be received (for instance, “*application/json*”, “*text/plain*”, “*application/xml*”). When receiving the response from a request, it can be verified if the content type of the response matches the one defined in the specification. The content type coverage metric measures how many valid content types were received from the one defined in the specification and it represents level TCL3 [7] of coverage. The results obtained for the “College football data” API are presented in Figure 13.

In the selected API, the total number of specified content types is 42. When using all the parameters (both *Max Parameter Coverage* and *Max Parameter Coverage and Partial Tests*), an increase from 38 to 40 content types covered can be observed. In the case of the content type coverage metric, the enabling of the partial tests generation feature (*Partial Test Generation* and *Max Parameter Coverage and Partial Tests*) does not impact the results. 

#### 5.1.8. Response Body Property Coverage

In some instances, the API definition may include the structure of the expected response body based on the content type in the operations response. For example, the operation *GET* for the path */metrics/wp/pregame* has a total of eight response body parameters, such as *gameId*, *season*, *homeTeam*, *week*, etc. 

**Figure 14:**
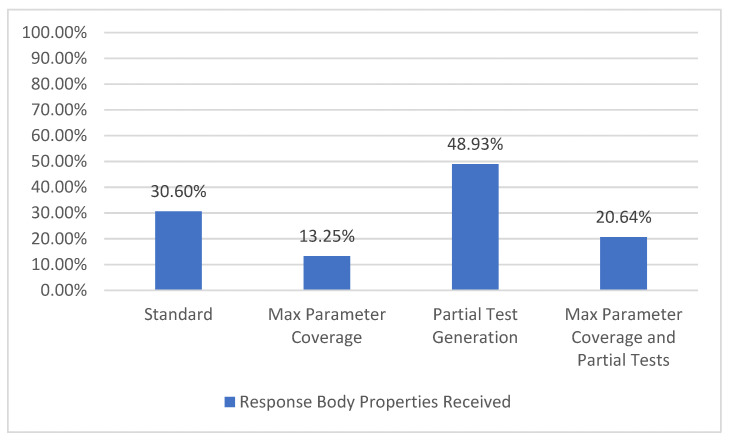
Response body property coverage.

Using this information, we may compute how many of the mentioned response body properties were actually received. This represents the response body property coverage metric (Figure 14), which has the strongest output coverage criteria TCL6 [7] as defined by Figure 2.

For the selected API, there are a total of 562 response body parameters. The configurations with partial tests (*Partial Test Generation* and *Max Parameter Coverage and Partial Tests*) lead to an increase in the number of received response body parameters because of the valid examples provided by the user. On the other hand, when using all the parameters (*Max Parameter Coverage* and *Max Parameter Coverage and Partial Tests*), a decrease in the percentage can be observed, which is attributed to the fact that most parameters have a role in filtering the response. Thus, using more parameters denotes getting a more filtered response with potentially less response body parameters. 

It should be noted that some APIs may or may not provide the response part for an operation or may provide only a part of the information. In those cases, depending on what information is available, it may not be possible to generate some of the following metrics: status code coverage, content type coverage, response body property coverage. 

#### 5.1.9. Performance Metric

**Figure 15:**
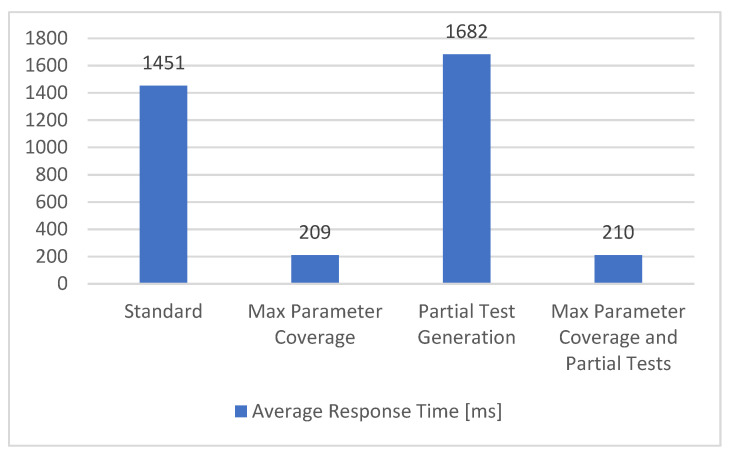
Performance metric.

To measure the performance of the API, we propose to measure the average time needed to receive the response for every request that is sent. In our solution, this average response time is represented by the performance metric and the obtained results are described in Figure 15.

In the configurations that use all the parameters (*Max Parameter Coverage* and *Max Parameter Coverage and Partial Tests*), a noticeable decrease in the time needed for the request to be completed could be observed. The reason is that a lot of parameters have the role of filtering the response, resulting in shorter responses and reduced average response times. The configurations that have partial tests do not present a significant change in the overall durations. As the performance metric can be affected by outside sources, such as network connectivity and latency, ideally, the metric should be measured on the APIs side.

### 5.2. Second Case Study: Testing a Set of 30 APIs

To further evaluate our proposed solution, we applied the same procedure as described in the previous case study to a total of 30 APIs. Using the services of APIs.guru (https://apis.guru/browse-apis/, accessed on 6 August 2021), we filtered out all APIs that did not have the OpenAPI version 3.0 or above and obtained about 800 APIs. Additionally, we eliminated the APIs that require a security key/API key/authentication (any APIs that had some form of security and needed credentials to be tested) and we obtained about 100 APIs. After filtering out the ones that had faulty specifications, we gathered a total of 30 APIs that ranged from simple (APIs with one or two paths) to complex (30 paths or above). The following list contains the names of all the 30 mentioned APIs that have been considered: “papiNet API CWG” API, “Corrently” API, “Data & Search team at UK Parliament” API, “JSON storage” API, “XKCD” API, “HackathonWatch” API, “shipstation” API, “topupsapi” API, “Geomag” API, “Gravity” API, “NSIDC Web Service Documentation Index” API, “BikeWise v2” API, “ODWeather” API, “WorkBC Job Posting” API, Atmosphere” API, “Space Radiation” API, “NeoWs—(Near Earth Object Web Service)” API, “Domains RDAP” API, “API Discovery Service” API, “WikiPathways Webservice” API, “httpbin.org” API, “OSDB REST v1” API, “RiteKit” API, “Aviation Radiation” API, “eNanoMapper database” API, “Tweets and Users” API, “RAWG Video Games Database” API, “College Football Data” API, “Rat Genome Database REST API” API, “VocaDB” API.

The coverage metrics mentioned in the previous section were computed for the entire set of 30 APIs by running our proposed solution using the four different configurations. The total number of test cases that were generated for each of the four configurations is: Standard—1222 test cases; Max Parameter Coverage—3785 test cases; Partial Test Generation—1225 test cases; and Max Parameter Coverage and Partial Tests—3807 test cases.

In Figure 15 we present the aggregated results obtained from evaluating our solution over the set of 30 APIs. The results are presented as the median for each metric evaluated in the aforementioned scenarios.

**Figure 16:**
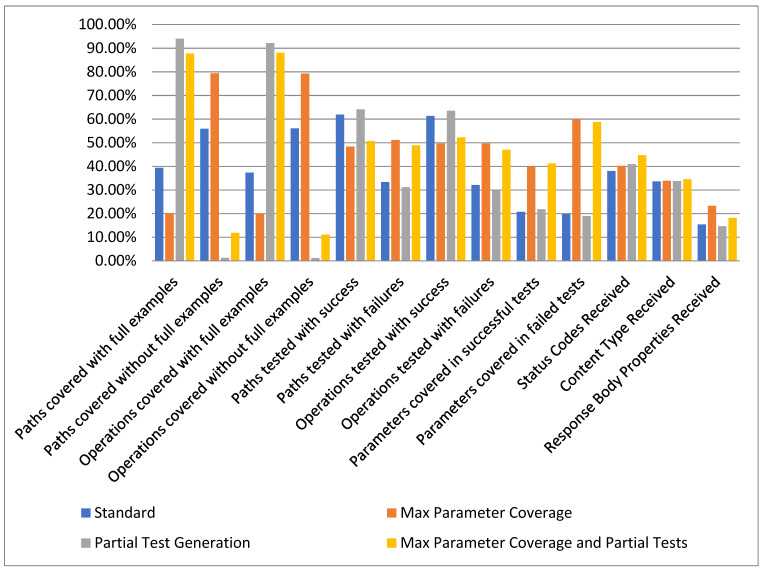
Overview statistics.

Given the overall results presented in Figure 16, we consider that our approach to provide the ability for the user to input examples has a significant positive effect. This can be observed in most of the evaluated metrics by comparing the *Standard* configuration with the *Partial Test Generation* configuration or comparing *Max Parameter Coverage* with *Max Parameter Coverage and Partial Tests*.

It can also be observed that the total number of requested parameters over the span of testing 30 APIs is not significantly large. In our case, it would average between four and twelve parameters per API, a value that requires a small amount of effort needed from the user.

## 6. Conclusions

The main purpose of our research was to provide a novel method to automate the testing of REST APIs based only on their OpenAPI 3.x specification. Using the method suggested in [4] as a starting point, our approach improves the test generation process by allowing minimal user interaction to provide examples for the parameters of the API operations. Furthermore, we expand the type of statistical results accompanying API testing to help gain a more in-depth analysis of each of the tested API.

One of the main advantages of our approach is its ease of use. The only things needed to test a given API is the URL or a file containing its API specification in OpenAPI 3.x format, and the parameter examples provided by the user depending on the selected configuration type. As mentioned previously, the user can easily change the configuration that determines how the tests are generated. Thus, the user can choose whether to use all the parameters in the API or to just use the required ones, as well as choosing whether additional examples should be expected from the user. Since these two options are independent from one another, it generates four possible configurations as described in Section 4. While the obtained coverage metrics provide a complex overview of the aspects tested over the API, our approach also provides a non-functional performance metric regarding the average time that is needed to run the chosen configuration of an API.

One of the disadvantages of our approach, and of the approaches that generate test cases exclusively based on API specifications in general, is that in the actual implementation of the API there are some interdependencies that cannot be extrapolated from the API definition alone. One such example would be the need to know an existing object in the API database before being able to perform a successful *PATCH* operation on it. Another downside is that, due to the way the average time performance metric is calculated, it is not guaranteed that there is no interference from external factors. Ideally, such a metric should be calculated directly on the API’s server side to avoid the inherent network latencies that may affect the results. Unfortunately, this cannot occur without having full access to the server.

Based on the current implementation which separates the parsing of the API and the test generation and execution, possible future improvements could include extending the number of specification languages that can be interpreted. Starting from the current performance metric, we could extend our implementation to also perform the load testing. This new functionality should simultaneously perform a series of requests to an API and monitor if the response time presents a noticeable difference. Another possible improvement would be the option to export the generated test cases in one or more testing frameworks, such as JUnit or Serenity.

In our approach, besides the fully automatic test case generation, we provide an option for the user to guide the test case creation process by incorporating the user’s expertise and the particularities of the context in which the API testing is performed. The software implementation is modular, the API specification parsing being separated from test generation and execution, which allows future extensions of specification languages that can be interpreted. The experiments proved the effectiveness and practicability of our method, while the wide range of coverage and performance metrics that are provided facilitate an in-depth analysis of the API testing process.

## Data Availability

Not Applicable.
